# Occurrence of Halogenated Pollutants in Domestic and Occupational Indoor Dust

**DOI:** 10.3390/ijerph17113813

**Published:** 2020-05-27

**Authors:** Giulia Simonetti, Patrizia Di Filippo, Carmela Riccardi, Donatella Pomata, Elisa Sonego, Francesca Buiarelli

**Affiliations:** 1 Department of Chemistry, Sapienza University of Rome, 00185 Rome, Italy; giulia.simonetti@uniroma1.it (G.S.); francesca.buiarelli@uniroma1.it (F.B.); 2 Dipartimento innovazioni tecnologiche e sicurezza degli impianti, prodotti e insediamenti antropici, Istituto Nazionale per l’Assicurazione contri gli Infortuni sul Lavoro (INAIL), 00143 Rome, Italy; p.difilippo@inail.it (P.D.F.); ca.riccardi@inail.it (C.R.); d.pomata@inail.it (D.P.)

**Keywords:** multiclass analysis, halogenated flame retardants, emerging pollutants, indoor settled dust, environmental exposures and health, occupational health, preventive measure

## Abstract

The occurrence of halogenated organic pollutants in indoor dust can be high due to the presence of textile, electronic devices, furniture, and building materials treated with these chemicals. In this explorative study, we focused on emerging organic pollutants, such as novel brominated flame retardants (nBFRs) and some perfluoroalkyl substances, together with legacy polychlorinated biphenyls (PCBs) and polybrominated diphenyl ethers (BDEs) in settled dust collected in houses and workplaces such as one office and two electrotechnical and mechanical workshops. The total contribution of the investigated pollutants was lower in house and in office dusts except for few nBFRs (such as bis (2-ethylhexyl)-3,4,5,6-tetrabromo-phthalate at a concentration of 464.5 ng/g in a house and hexachlorocyclopentadienyldibromocyclooctane at 40.4 ng/g in the office), whereas in electrotechnical and mechanical workshops a high incidence of PCBs, BDEs, and nBFRs occurred (for example, BDE 209 at a concentration of 2368.0 ng/g and tetrabromobisphenol A at 32,320.1 ng/g in electrotechnical and mechanical workshops). Estimated daily intakes were also calculated, showing that domestic and occupational environments can lead to a similar contribution in terms of human exposure. The higher exposure contribution was associated to nBFRs, whose EDIs were in the range of 3968.2–555,694.2 pg/kg bw/day. To provide a complete view about the indoor contamination, in this investigation, we also included polycyclic aromatic hydrocarbons (PAHs) and their oxygenated and nitrated derivatives. Definitely, dust collection represents a simple, fast, and cost-effective sampling and dust contamination level can be a useful indicator of environment healthiness. Besides, the presented method can be a smart tool to provide a time and money saving technique to characterize 99 pollutants thanks to a single sample treatment.

## 1. Introduction

Flame retardants (FRs) are chemicals commonly added to many products to reduce the flammability of that product by increasing the ignition point and curtailing the spread of a fire. However, several studies showed that many of these compounds had adverse health effects, such as endocrine disruption, carcinogenic effects, immunotoxicity, and reproductive toxicity [1]. Among the organic FRs, polychlorinated biphenyls (PCBs), produced since 1930, were used in thousands of consumer products. Some of them, called dioxin-like, have chemical-physical characteristics that make them similar to dioxins and furans, causing therefore the same toxicological effects. European countries have started to apply measures to prohibit equipment containing PCBs since 1986, but the complete elimination of PCBs has not been achieved. In addition, brominated flame retardants (BFRs) including polybrominated diphenyl ethers (BDEs) were widely used as additives in industrial materials [2]. The structural similarity of BDEs with thyroxine, the main thyroid hormone, induces interference with the endocrine system [3]. An adverse effect in the neurological development of exposed children has been shown [4] and association between high concentrations of some BDEs in adipose tissues and the onset of cancer was also suggested [5]. Therefore, the Stockholm Convention of 2009 banned production of many of these substances [6,7].

Due to the bans on the use of the aforementioned FRs, but owing to their essential role, new brominated flame retardants (nBFRs) have been introduced on the market. For these alternative chemicals, little or no toxicity data exist; therefore, there is no scientific evidence that these products are safer than the banned ones. Indeed, because of physico-chemical properties similar to the legacy FRs, a similar toxicological behavior would be expected, including the endocrine toxicity, carcinogenicity, and environmental fate [8]. Among the main nBFRs in use, we investigated the chemicals listed in Table 1 and described below.

Often data were insufficient to provide a clear picture of these chemicals; however, some studies were available about toxicity and health effects. TBBPA, the most widely produced brominated flame retardant in the world, was related to effects on reproductive and nervous system development, including brain and thyroid function. TBP, the most widely produced brominated phenol, has been described as a disruptor of hormonal regulation [14]. Among tribromophenoxy FRs, BTBPE, DPTE (or TBP-DBPE), and ATE (or TBP-AE), extensively used in the US and China, may exhibit the same toxicity mechanism of BDEs for their structural similarity [15]. Although HBB is not teratogenic or fetotoxic, it is metabolized in rat liver into toxic molecules [16]. PBBA shows the ability to bind and activate estrogen and androgen receptors, but data are insufficient to provide a clear picture [17]. TBCO, used in replacing HBCD (hexabromocyclododecane), shows similar potentials for bioaccumulation, persistence, and long-range atmospheric transport as BDEs and HBCD [18]. In vitro studies about TBECH have shown the ability to bind and activate the androgen receptor (hAR) with high affinity. Even low-level exposure may cause behavioral, functional, and developmental disorders [19].

Perfluorinated alkylated substances (PFAS) represent another emerging pollutant of increasing interest added to many materials to increase their resistance. PFAS have been produced since 1920; in particular, perfluoroctane acid (PFOA) and perfluoroctane sulfonic acid (PFOS) were extensively used; however, many in vivo and in vitro studies have suggested that exposure to PFOS may lead to adverse effects on human health, such as hepatotoxicity, neurotoxicity, reproductive toxicity, immunotoxicity, thyroid disruption, cardiovascular toxicity, pulmonary toxicity, and renal toxicity [20,21,22]. Hence, PFOS and its salts were classified as persistent organic pollutants (POP) by the Stockholm Convention in 2009; these compounds are still found in the environment, because of their persistence, and because of degradation of molecules alternatively used such as fluorotelomer alcohols (FTOHs) [23].

In this study, we investigated the presence of these pollutants in indoor environments. In fact, electric and electronic components, insulation wires, cables, domestic appliances, furniture, upholstery, mattresses, and carpets can be a source of FRs and PFAS. Then, in indoor environments, such as houses and workplaces, the concentration of these compounds can be high. The settled dust can be rich in FRs both because of the presence in plastic particles abraded from product surfaces, and because of adsorption on dust of volatilized FRs, released from these products. The emission can vary based on product source, kind of released FR, because of the compound-specific release time and rate, determined by its physical properties (FRs with higher vapor pressures evaporate faster even at room temperature) [24]. Hence, a passive deposition onto the surface of the buccal, nasal, and ocular mucous membranes causes indirect ingestion of dust. In addition, dermal adsorption is also an important human exposure pathway. It is noteworthy that the sampling of settled dust is rapid and cost effective compared to air sampling. Despite the difference in dust and air contamination, a simple and rapid chemical characterization of indoor dust, characterized by cost-effective sampling, can be a useful indicator of environmental healthiness. In our previous studies, FRs levels in air and dust of an electrical and electronic waste treatment facility were obtained through an improved analytical method [25,26]. In this explorative study, the previous method was optimized and extended to a new pollutant class (PFAS) and to some new BDEs and nBFRs, allowing the monitoring of four different classes of pollutants in domestic and occupational indoor dust. For this purpose, the samples were collected in two houses (apartments), one office, and two electronic and mechanical workshops. In addition, the polycyclic aromatic hydrocarbons (PAHs), their oxygenated derivatives (oxy-PAHs), and nitro-polycyclic aromatic hydrocarbons (nitro-PAHs) were included for their ubiquity and health concern, in perspective of a cumulative exposure assessment to environmental pollutants. Most of the studies have generally focused on a single pollutant class; in contrast, this study, by monitoring a total of 99 compounds, represents an important goal to improve knowledge of human exposure in contaminated indoor environments.

## 2. Materials and Methods

### 2.1. Standard 

TBPH, BTBPE, PBBA, all the PCBs, and all the BDEs standards were purchased from Wellington Laboratories (Ontario, Canada); ATE, PBEB, HBB, PFTeDA, FOSA, all PAHs, PAH derivatives and mass labeled PCBs, and PFOA, used as internal standards, were purchased from Sigma Aldrich (Milan, Italy); and PFOA, PFOS, PFBS, TBB, TBECH, TBBPA, DPTE, TBCO, and TBP were obtained from AccuStandard Inc. (Connecticut, USA). Stock standard solutions were prepared from solid analytes by dissolving each compound in toluene (1 mg/mL) or from purchased standard solution (50 μg/mL) sealed with screw-caps and stored at −20 °C in amber glass vials.

### 2.2. Chemicals, Reagents and Other Materials

MS grade n-hexane, toluene, ethyl acetate, acetonitrile, methanol, and isopropyl alcohol were purchased from Sigma-Aldrich S.r.l. (Milan, Italy). Water purified by a Milli-Q^®^ Integral system (Merck KGaA, Darmstadr, Germany) (no PFAS containing polymers) was used. Florisil sorbent, 30–60 mesh was purchased from Sigma Aldrich S.r.l. (Milan, Italy). Regenerated cellulose (RC) filters (15 mm × 0.22 µm) with polypropylene housing were from Phenomenex (Torrance, CA, USA).

### 2.3. Dust Sample Collection

The dust samples were collected during Summer 2019 in five different sampling sites located in different areas of Rome, namely two domestic environments (D1 and D2), one office (D3), and two electrotechnical and mechanical workshops (D4 and D5), using a household vacuum cleaner in which a single vacuum bag (previously tested for the presence of target contaminants) was fitted for each sampling site for the entire sampling period. During the sampling, no area or time was defined to collect a significant amount of dust. The dust samples were homogenized and sieved (63 μm, Giuliani, Torino, Italy) before use to remove all the impurities (hairs, crumbs etc.) or other non-dust parts; the smaller fraction was finally stored at −20 °C until analysis to reduce the possible degradation of the samples. 

### 2.4. Extraction Procedure and Analysis 

A single sample treatment was optimized for the extraction of all the investigated compounds using accelerated solvent extraction (ASE) technique. In-cell clean-up was used to perform organic pollutant extraction according to other studies [26,27,28], as well as to perform a simultaneous extraction and purification by ASE. The analyses were carried out using mass spectrometric detectors coupled with high performance liquid chromatography or gas chromatography (HPLC-MS/MS or GC-MS). 

An amount of 100 mg of dust spiked with an IS (internal standards) mixture was extracted by an accelerated solvent extractor ASE 200 (Dionex, Sunnyvale, CA, USA). ASE cells were fitted with florisil (500 mg) sorbent to simultaneously obtain extraction and purification of the samples. The extraction was carried out with n-Hexane (one cycle) followed by ethyl acetate (one cycle) and finally isopropyl alcohol/methanol 90/10 (v/v) (one cycle) at 100 °C and 1500 psi. The use of this extraction solvents allowed the extraction of all pollutant classes under investigation from the sample. The extract was split into two equal aliquots and evaporated using an Evaporator SE 500s-Dionex (Dionex, Sunnyvale, CA, USA), quantitatively transferred to conical vials, and carefully evaporated to dryness under a gentle nitrogen stream. Finally, the extracts were re-dissolved with 50 μL of toluene for GC-MS analyses and 50 μL of acetonitrile for HPLC-MS-MS containing the internal standards. The analyses of 21 PCBs, 11 BDEs, 13 nBFRs, 20 PAHs, and 28 PAH derivatives (oxy-PAHs and nitro-PAHs) were performed in GC-MS. An HP 7890-B gas chromatograph fitted with an HP 7693 autosampler and coupled with an HP 5977B single quadrupole mass-selective detector (Agilent Technologies, Palo Alto, CA) was used for GC-MS analysis both in electronic ionization (EI) and in negative chemical ionization (NCI). The instrument was tuned using the software autotune procedure (Agilent MSD Chem Station D.01.00 software) (Agilent Technologies Italia S.p.A., Cernusco sul Naviglio MI, Italy) and selective ion monitoring (SIM) was used in both MS configurations. The injector temperature was set at 280 °C and the samples (1 µL) were injected in splitless mode. The helium carrier gas was set at a constant flow rate of 1.0 mL/min. Analytical methods were described in detail in previous studies [25,26,29,30,31] with the inclusion of PBBA and TBP and implemented with other BDEs. On the other hand, the analyses of PFAS, TBBPA, and BDE 209 were carried out using HPLC-MS/MS. The HPLC 1260 Infinity II system (Agilent Technologies Italia S.p.A., Cernusco sul Naviglio MI, Italy), fitted with an autosampler (injection volume 8 μL) Agilent1260 G7129A, was coupled to a triple quadrupole mass spectrometer API 2000 (AB SCIEX S.r.l. Forster City, CA, USA). The apparatus was coupled with an electrospray ionization (ESI) source set at 350 °C, for PFOA, PFOS, PFBS, PFTeDA, FOSA, and TBBPA analysis. The chromatographic separation was carried out with a Gemini C_18_ 3 µm, 150 × 2 mm (Phenomenex) column. The flow rate was set at 0.2 mL/min and the column temperature at 40 °C. The mobile phases A and B were water and methanol, respectively, both containing 5 mM ammonium formate. The following gradient elution was used: 70% B increase to 100% in 15.00 min, from 15.01 to 29.99 min keep isocratic, at 30.00 min return to 70% B, and finally re-equilibrate the column at 70% B for 30 min. The injection volume was 5 μL. The BDE 209 analysis was carried out using the atmospheric pressure chemical ionization source (APCI) in the same chromatographic condition without ammonium formate in mobile phases. The analyses were carried out in negative polarity in multiple reaction monitoring acquisition.

### 2.5. Data Quality

The analytical performances were assessed to verify whether the method fits for the purpose. In absence of a standard reference material, to get the quality assurance, both a procedural blank and a quality control (QC), obtained by spiking to a blank sample a standard mix solution at a LOQ concentration, were processed in each analytical session. Particular attention was paid to verify the absence of contamination given by plastic devices, glassware and solvents and the QC fulfilled the requirements of precision (RSD ≤ 15%) and trueness (expressed as relative error, E% ≤ 20). The linearity was assessed with R^2^ of standard solvent calibration curves. Instrumental limit of detection (LOD) and quantification (LOQ) were calculated as reported by Buiarelli et al. [25] and described in Section 3.1. The extraction recoveries were calculated with triplicate analyses in two analytical sessions by the ratio of post-extraction versus pre-extraction spiked blank samples. Intra- and inter-day repeatabilities of the method were calculated by replicate analyses of a multistandard solution injected five times in the same day and in five non-consecutive days. Intra- and inter-day repeatability were expressed as (RSD); values within 15% can be considered suitable for multi-analyte methods.

### 2.6. Quantitative Analysis

Different calibration curves (matrix-free and matrix-matched) were built, both in HPLC-MS/MS and in GC-MS. The standard solvent (matrix-free) calibration curves were prepared using increasing concentrations of analytes and a constant concentration of internal standards (IS). Taking into account the different instrumental sensitivities, in HPLC-MS/MS, concentrations ranged 50.0–1000.0 ng/mL, whereas in GC-MS they ranged 0.5–800.0 ng/mL. The ratio of analyte area to IS area was plotted versus analyte concentration and a linear regression was obtained for each analyte in the linearity range. The quantitative analyses were carried out using the matrix-matched curve, as described by Buiarelli et al. [25]. Briefly, five aliquots of 100 mg of the least contaminated dust in this study (D3) were spiked with IS and pollutant standard solutions at increasing concentrations prior to the extraction. The linear plot of the analyte/IS area ratio versus standard addition concentration was drawn. The analytes concentrations in the office dust were estimated using the intercepts on the abscissa. The other dust samples were quantified using this curve translated to the origin, by subtracting to each point the endogenous contribution. Then, the results were automatically corrected for extraction recoveries and matrix effect. Due to the chromatographic coelution of PCB 77 with PCB 110 and PCB 170 with PCB 190, given concentrations express the sum of these compound pairs.

## 3. Results and Discussion

### 3.1. Method Performances

Matrix-free calibration curves showed linearity always associated to R^2^ > 0.995, and matrix-matched calibration curves provided R^2^ between 0.980 and 0.999. Method LOQ ranged 0.5–9.5 ng/g for PCBs; 1.0–14.0 ng/g for BDEs, except for BDE 209 value equal to 250.0 ng/g; 1.2–248.2 ng/g for nBFRs; and 5.0–157.0 ng/g for PFAS. The sample preparation (Section 2.4) allowed for the simultaneous extraction and purification of a broad and heterogeneous group of compounds Considering the number of analytes monitored, total recoveries ≥40% were acceptable if associated to an RSD ≤ 20. A lower extraction selectivity affects the quantitative analysis, due to the higher background and matrix effect. Nevertheless, recoveries above 50% were detected for most compounds (97%), with recoveries ranging 85–115% for 72% of the analytes. The recovery obtained were in the range 70–96% for PCBs, 86–106% for BDEs, 41–116% for nBFRs, and 57–97% for PFAS. The intra- and inter-day repeatabilities expressed as RSD were below 10% and 12%, respectively.

### 3.2. Indoor Sample Analysis

Table 2, Table 3 and Table 4 show the detected concentrations for PCBs, BDEs, and nBFRs, respectively. In the tables, n.d. indicates not detected compounds, while values between LOD and LOQ were considered positive and reported as <LOQ. Columns 2–6 display the specific values relative to each sampling site (D1–D5) of the investigated compounds, and the last row shows the total contribution associated to the same pollutant class expressed as a sum (ΣBDEs, ΣnBFRs, and ΣPCBs). Columns 7 and 8 present the pollutant-specific averages of domestic dusts (D1 and D2) and workplaces dusts (D3–D5), respectively, to easily compare the two different scenarios.

PCB 99, PCB 151, and PCB 169 were not detected in any sample and only PCB 180 was detected in all samples. The total contribution of PCBs contamination, expressed as the sum of homologs concentration (ΣPCBs), was higher in workplaces than in domestic dust. In particular, D5 values result about four times higher than D4, possibly because of the larger workshop size and older devices stored in D5 workshop. D4 and D5 results were also above the concentration found for similar workplace category in other studies [26,34]. In the absence of a specific limit for PCBs in dust, we compared the obtained concentrations with the value reported from EPA (Environmental Protection Agency) for the soil in Regional Screening Level Tables, equal to 0.22 μg/g [35]. In D5, PCB concentration (0.55 μg/g) exceeded more than twice the EPA value. Furthermore, we monitored eight dioxin-like PCBs (DL PCBs), due to the health concern about these substances. ΣDL PCBs was 32.2, 40.0, 150.3, 24.6, and 298.7 ng/g for D1–D5, respectively. The percentage of ΣDL PCBs concentrations versus total PCBs concentrations (ΣPCBs) was around or above 50% for all samples except for D4, indicating that these harmful compounds can be still found.

As can be seen in Table 3, BDE 28, BDE 85, and BDE 197 were not detected in any sample, whereas BDE 47 and BDE 99 were found in all samples. Similar to PCBs, ΣBDEs was higher in electronic and mechanical workshops (D4 and D5). BDE 209 is the most widespread BDE and it was found in high concentrations in several studies [36,37,38]. In our work, BDE 209 was detected only in one sample (D5) and it represented the most concentrated (2368.0 ng/g) compound among BDEs investigated. As shown in Table 3, the values obtained for each congener were within the contamination range reported in other studies, except for BDE 100 that resulted two and five times higher than in the studies of Fromme [32] and de la Torre [33], respectively.

Among nBFRs, the most concentrated compound was TBBPA in D4, detected at a concentration of 32,320.1 ng/g. In this study, TBBPA was not detected in the other samples, possibly because of the high LOD of our method (124.1 ng/g). D4 concentration was two orders of magnitude higher compared to TBBPA concentrations in other indoor dust studies [9,36,39]. However, high TBBPA concentrations were found in workplaces where similar activities were carried out, such as dismantling of electronic devices in recycling plants [40]. The high contamination level of TBBPA in D4 can be explained considering the workers practice to remove the dust stored inside the devices before repairing them. In fact, TBBPA, added in many resins used in plastic casings of electronic devices [9], was found in high concentrations in computer cooling fan parts. Many FRs are frequently added in those parts because of the risk of overheating due to the accumulated dust between the plug and the wall outlet [41]. About the other 12 nBFRs, BTBPE average values were higher than other studies [32,33,38,42] and HBB showed average values about ten times higher than the literature values [33,36,38,42]. TBP and TBPH were detected in all samples, in agreement with other studies (100% of samples incidence) [32,43]. TBP values were 788.4 ng/g in D4 and 464.5 ng/g in D1, while TBPH was higher in D1 and D2 (182.9 and 464.5 ng/g respectively). TBPH had a large commercial use as Firemaster 550 mixed with TBB (ratio of 1:4) but, in the present study, a TBPH concentration higher than TBB was found, in accordance with other studies [42,44], due to the faster transfer of TBB than TBP. Nevertheless, not accounting for TBPH contamination, the total concentration (ΣnBFRs) results higher in electronic and mechanical workshops (D4 and D5) than in houses and office dusts.

Regarding the PFAS, PFOA was the only detected compound with a concentration lower than the LOQ (119.3 ng/g) in both D4 and D5 samples, while PFBS, PFOS, PFTeDA, and FOSA were not detected in any samples. 

To have a complete view of the indoor pollution and in the perspective of a cumulative exposure assessment to environmental pollutants, thanks to the versatility of our method, the samples were also analyzed to determine the PAHs. Sums of PAHs (ΣPAHs and Σ(nitro-PAHs + oxy-PAHs)) were calculated. ΣPAHs were 417.2, 5247.9, 1720.2, 4917.6, and 7601.4 ng/g for D1–D5, respectively, while Σ(nitro-PAHs + oxy-PAHs) were 733.1, 495.7, 145.5, 3447.2, and 5110.9 ng/g for D1–D5, respectively. PAHs and PAH derivatives were generated from outdoor combustion processes. The results assess that indoor contamination strongly depends on the outdoor environment. It should be noted that the D1 sample, collected in a suburban apartment, showed less contamination for PAHs and PAH derivatives than the other samples, collected in a central area of the city of Rome, strongly characterized by motor vehicle traffic.

### 3.3. Estimate of Human Exposure

To evaluate the human exposure, EDI (estimated daily intake) was calculated for PCBs, PBDEs, and the main nBFRs detected. EDIs were calculated for each environment separately rather than using the domestic and workplace averaged concentrations, since they were not considered representative because of the small number of samples treated in this preliminary study. Settled dust can be unintentionally ingested via passive deposition onto the surface of the buccal, nasal, and ocular mucous membranes as well as hand-to-mouth activity. In addition, dermal adsorption can also contribute to the exposure. These exposure contributions were calculated as EDI_ingestion_ and EDI_dermal_ using the following formulas [33]:

EDI_ingestion_ = (C_dust_ x IR_dust_ x AF_gastro_ x EF)/BW (1)

EDI_dermal_ = (C_dust_ x DAS x ESA x AF_dermal_ x EF)/BW (2)

The used parameters are summarized in Table 5 and Table 6. C_dust_ is the experimental pollutant concentration, IR_dust_ represents the amount of unintentional dust ingestion rate per day, AF_gastro_ is the percentage amount of pollutant adsorbed gastrointestinally, and the indoor exposure fraction (EF) represents the percentage of time spent in indoor environments in a day;, hence, different values were used for domestic dusts (D1 and D2) and workplace dusts (D3–D5). For dermal adsorption calculation, the dust adherent to skin (DAS) and the dermal adsorption factor (AF_dermal_) were used. AF_dermal_ has a specific parameter for each pollutant (Table 5 and Table 6). It was not available for some nBFRs; thus, according to Tay et al. [45], factors associated to other similar compounds, considering the molecular weight and the number of bromines, were chosen. In particular, we used the BDE 99 value for PBBA. Because of the absence of children in the environment studied, only adult values were used for body weight (BW) and exposed skin area (ESA). To avoid underestimation, we preferred the worst-case scenario values when different values could be used.

Table 7 shows the values obtained. For each environment, EDI_ingestion_ and EDI_dermal_ were summed and are reported as ΣEDI_(ingestion+dermal)_. However, it is worth noting that the major indoor dust exposure was due to EDI_ingestion_, which resulted 3–4 orders of magnitude higher than EDI_dermal_ in all the cases (data not shown). In addition, the overall contribution of each pollutant class as ΣEDI ΣPCBs, ΣEDI BDEs, and ΣEDI nBFRs are reported. The total estimated ΣEDI_(ingestion+dermal)_ of each compound was compared with the reference dose available (RfD, Table 7). RfD was the assumed daily exposure related to no appreciable risk of deleterious effects during a lifetime. In particular, the ΣEDI _(ingestion+dermal)_ for PCBs were in the range of 1976.3–7687.3 pg/kg bw/day, with the mechanical workshop (D5 dust) giving the highest exposure. However, the ΣEDI_(ingestion+dermal)_ for PCB resulted always lower than 6% of RfD reported. The ΣEDI_(ingestion+dermal)_ for BDEs were in the range of 2103.8–430,05.0 pg/kg bw/day; in this case as well, the mechanical workshop (D5) was related to the highest exposure. For the other workplaces, despite higher concentrations revealed in dust, the ΣEDI_(ingestion+dermal)_ resulted less than or equal to domestic exposures, because of the different EF factor used. In none of the environments, the ΣEDI_(ingestion+dermal_) was higher than 3% of RfD. Finally, the highest exposure contribution was associated to nBFRs; the ΣEDI_(ingestion+dermal)_ values were in the range of 3968.2–555,694.2 pg/kg bw/day. The electronic repair workshop (D4) gave the highest value, but, also in this case, the other workplaces led to lower or similar exposures compared to domestic environments. In any case, the ΣEDI_(ingestion+dermal)_ related to nBFRs resulted in less than 0.05% of RfD available.

To sum up, the ΣEDI_(ingestion+dermal)_ values obtained were several orders of magnitude lower than RfD, indicating that the dust contamination in the environment investigated was not of health concern. However, the high concentrations of some FRs in both domestic and occupational environments suggest complementary air sampling of those compounds to have information about inhalation exposure as well.

Even though many data are available about human exposure to legacy PCBs and BDEs, there is still a lack of information about nBFRs exposure. The accurate environmental distribution and human exposure of emerging pollutants such as nBFRs, together with toxicological data, are fundamental findings for risk assessment by authorities and for proposing regulation directives. This explorative study may be the basis for further studies, applying the proposed methodology to an extensive number of samples. 

## 4. Conclusions

In this study, we monitored 21 PCBs, 12 BDEs, 13 nBFR, and 5 PFAS together with 20 PAHs and 28 PAH derivatives (nitro-PAHs and oxy-PAHs) in settled indoor dust. A unique sample treatment was optimized for simultaneous extraction of the 99 pollutants, obtaining acceptable analytical performance for such an explorative study. Domestic and occupational environments were considered, namely the dusts from two homes, one office, and two dusts of electronic and mechanical workshops were analyzed. The electronic and mechanical workshops showed the highest contamination for legacy FRs as PCBs and BDEs. The contamination by nBFRs was heterogeneous; in particular, TBBPA and TBP in D4 and TBPH in D2 were detected in higher concentrations. The preliminary investigation of PFAS showed only for PFOA detectable concentrations above LOQ in mechanical and electronical workshops. Because of the PFOS and PFOA phase out from the market, analysis of alternatively used PFAS will be the next step in future studies. Contamination by PAHs was, as expected, stronger in the samples collected in the central area of Rome than in D1 sample collected in suburbs area.

To assess contaminated dust contribution to human exposure, we calculated pollutants EDI as the sum of unintentional ingestion and dermal adsorption pathways. Despite the higher pollutant concentrations in workshops, EDI by PCBs and BDEs showed a similar contribution in both domestic and workshop scenarios, because of the different amounts of time spent in these environments (EF factor). Higher EDI values were related to nBFRs in all samples except for D5 environment. The EDI values were several orders of magnitude below the RfD available, suggesting that the exposure to dust may not pose a health risk in the investigated buildings. The significant concentrations of some FRs found in this explorative survey indicate that both domestic and occupational scenarios should be monitored. In addition, air concentrations of those contaminants may useful to fully characterize the indoor pollutant distribution and exposure. The proposed indoor dust method, characterized by rapid and cheaper sampling, can be applied to more houses and different workplaces in further studies to delve into the topic about the indoor environment pollution, required to reduce contaminant exposure to humans.

## Figures and Tables

**Table 1:** Investigated nBFRs, their abbreviations, restriction and the main applications and uses.

Abbreviation	Nomenclature	Restriction	Application and Use
TBBPA	Tetrabromobisphenol A	✓ *	Additive in resins used in plastic casings of electronic devices and in printed circuit boards and in several types of polymers [9].
TBPH	bis (2-ethylhexyl)-3,4,5,6-tetrabromo-phthalate	X	Used in the foams of polyurethane as a mixture of TBB and TBPH (ratio about 4:1 in mass) commercially known as “Firemaster 550”.
TBB	2-Ethylhexyl-2,3,4,5-tetrabrombenzoate	X	Additive in foams of polyurethane.
TBP	2,4,6-tribromophenol	X	Added in polyurethanes plastic, resins and paper-based products and flame retardant intermediate [10].
BTBPE	1,2-bis(2,4,6-tribromophenoxy) ethane	✓ **	Additive used in acrylonitrile-butadiene styrene copolymers (ABS), high impact polystyrenes (HIPS), and in electronics.
DPTE	2,3-dibromopropyl-2, 4, 6-tribromophenylether	✓ **	Main component of the brominated flame retardant (BFR) Bromkal 73-5 PE.
ATE	Allyl-2,4,6-tribromophenyl ether	✓ **	Additive use in EPS and PS foam (both rigid and flexible foams).
HBB	Hexabromobenzene	X	Additive flame retardant in paper, textiles, electronics, and plastics and decomposition product of other FRs.
HCDBCO	Hexachlorocyclopentadienyldibromocyclooctane	X	Additive in plastics and polymers, especially in polystyrene [11].
PBBA	Pentabromobenzyl Acrylate	X	Used as monomer in dispersion polymerization process polyester and polystyrene.
PBEB	2,3,4,5,6-pentabromoethylbenzene	✓ **	Additive in circuit boards, textiles, wire coatings, and polyurethane foam [12].
TBCO	1,2,5,6–tetrabromocycloctane	X	Additive in plastics, paints and in the textile industry.
TBECH	1,2-dibromo-4-(1,2-dibromoethyl) cyclohexane	X	Additive in construction materials, electric cables, polystyrene-based insulation panels, plastics and adhesives.

* Legislative restrictions in Europe through the IPPC (Integrated Pollution Prevention and Control) Directive [13]. ** Chemicals listed as Low Production Volume (LPV) chemical in Europe.

**Table 2:** PCB values expressed as ng/g of dust, reported for each congener, in the different indoor environments (Columns 2–6). Columns 7 and 8 show the domestic dust average (D1 and D2) and workplace dust average (D3–D5). The reported values were associated to a method RSD of 15%.

Compound	D1 (ng/g)	D2 (ng/g)	D3 (ng/g)	D4 (ng/g)	D5 (ng/g)	Houses Average (ng/g)	Workplaces Average (ng/g)
PCB 77+110	^1^ n.d.	^1^ n.d.	150.3	^1^ n.d.	204.9		177.6
PCB 81	24.9	9.7	^1^ n.d.	19.5	^1^ n.d	17.3	9.8
PCB 99	^1^ n.d.	^1^ n.d.	^1^ n.d.	^1^ n.d.	^1^ n.d.		
PCB 101	^1^ n.d.	^1^ n.d.	37.4	^1^ n.d.	^1^ n.d		18.7
PCB 105	^1^ n.d.	^1^ n.d.	^1^ n.d.	^1^ n.d.	56.6		56.6
PCB 114	7.3	7.0	^1^ n.d.	^1^ n.d.	^1^ n.d.	7.2	
PCB 126	^1^ n.d.	^1^ n.d.	^1^ n.d.	^1^ n.d.	3.9		3.9
PCB 138	^1^ n.d.	11.0	10.2	31.2	83.4	11.0	41.6
PCB 146	<2.8	<2.8	^1^ n.d.	3.7	12.7		8.2
PCB 151	^1^ n.d.	^1^ n.d.	^1^ n.d.	^1^ n.d.	^1^ n.d.		
PCB 156	^1^ n.d.	4.7	^1^ n.d.	5.1	11.2	4.7	8.1
PCB 157	^1^ n.d.	8.5	^1^ n.d.	^1^ n.d.	8.8	8.5	8.8
PCB 167	^1^ n.d.	10.1	^1^ n.d.	^1^ n.d.	13.3	10.1	13.3
PCB 169	^1^ n.d.	^1^ n.d.	^1^ n.d.	^1^ n.d.	^1^ n.d.		
PCB 170+190	4.4	5.2	^1^ n.d.	13.8	24.7	4.8	19.3
PCB 177	^1^ n.d.	2.9	^1^ n.d.	6.0	17.6	2.9	11.8
PCB 180	8.7	12.7	5.0	33.0	65.1	10.7	34.4
PCB 183	18.3	17.4	^1^ n.d.	19.8	24.8	17.9	22.3
PCB 187	<1.4	<1.4	^1^ n.d.	8.2	19.0		13.6
^2^ ΣPCBs	63.6	89.3	203.0	140.4	546.1	76.4	296.5

Values between LOD and LOQ were considered positive and reported as <LOQ value. ^1^ n.d., not detected. ^2^ ΣPCBs is the sum of PCB concentrations.

**Table 3:** BDE values expressed as ng/g of dust, reported for each congener, in the different indoor environments (Columns 2–6). Columns 7 and 8 show the total average value of the domestic dust average (D1 and D2) and workplace dust average (D3–D5). The Column 2–8 values were associated to a method RSD of 15%. Results are compared with other studies (last two columns) reported as median (min.–max.) concentrations.

Compound	D1 (ng/g)	D2 (ng/g)	D3 (ng/g)	D4 (ng/g)	D5 (ng/g)	Houses Average (ng/g)	Workplaces Average (ng/g)	Fromme et al. [32] (ng/g)	de la Torre et al. [33] (ng/g)
BDE 28	^1^ n.d.	^1^ n.d.	^1^ n.d.	^1^ n.d.	^1^ n.d.				0.08 (<0.03–0.36)
BDE 47	13.8	25.4	12.1	42.1	38.3	19.6	30.8	11.7 (1.3–52.3)	2.74 (0.08–23.1)
BDE 49	^1^ n.d.	^1^ n.d.	^1^ n.d.	^1^ n.d.	^1^ n.d.				
BDE 66	^1^ n.d.	^1^ n.d.	53.8	^1^ n.d.	^1^ n.d.		53.8		(<0.02–0.40)
BDE 85	^1^ n.d.	^1^ n.d.	^1^ n.d.	^1^ n.d.	^1^ n.d.				0.19 (<0.01–3.7)
BDE 99	20.5	54.4	23.6	56.5	58.8	37.4	46.3	21.7 (1.0–84.1)	4.97 (0.11–46.7)
BDE 100	47.9	20.5	6.4	^1^ n.d.	51.9	34.2	29.2	3.5 (2.0–15.8)	0.65 (0.03–6.83)
BDE 153	^1^ n.d.	^1^ n.d.	^1^ n.d.	^1^ n.d.	^1^ n.d.		16.1	4.6 (2.0–20.5)	0.78 (<0.02–6.47)
BDE 154	^1^ n.d.	6.4	2.0	^1^ n.d.	9.0	6.4	5.5	2.5 (2.0–9.5)	2.59 (<0.04–25.3)
BDE 183	^1^ n.d.	12.4	12.5	134.4	74.3	12.4	73.7	27.9 (2.0–394)	1.11 (0.1–22.9)
BDE 197	^1^ n.d.	^1^ n.d.	^1^ n.d.	^1^ n.d.	^1^ n.d.				
BDE 209	^1^ n.d.	^1^ n.d.	^1^ n.d.	^1^ n.d.	2368.0		2368.0		232 (5.36–2470)
^2^ ΣBDEs	82.2	119.0	126.5	233.0	2600.3	100.6	986.6		

Values between LOD and LOQ were considered positive and reported as <LOQ value. ^1^ n.d., not detected. ^2^ ΣBDEs is the sum of BDE concentrations.

**Table 4:** nBFR values expressed as ng/g of dust, reported for each congener, in the different indoor environments (Columns 2–6). Columns 7 and 8 show the total average value of the domestic dust average (D1 and D2) and workplace dusts average (D3–D5). The Column 2–8 values were associated to a method RSD of 15%. Results are compared with other studies (last two columns) reported as median (min.–max.) concentrations.

Compound	D1 (ng/g)	D2 (ng/g)	D3 (ng/g)	D4 (ng/g)	D5 (ng/g)	Houses Average (ng/g)	Workplaces Average (ng/g)	Fromme et al. [32] (ng/g)	de la Torre et al. [33] (ng/g)
TBBPA	^1^ n.d.	^1^ n.d.	^1^ n.d.	32320.10	<248.2		32,320.1	44.1 (2.9–233)	
TBPH	182.9	464.5	64.5	<44	<44	323.7	64.5	20 (25–2274)	
TBB	12.0	11.7	^1^ n.d.	9.6	9.7	11.9	9.7	4.2 (<3.0–13.6)	
TBP	26.4	51.7	16.7	788.4	54.4	39.0	286.5		
BTBPE	^1^ n.d.	^1^ n.d.	17.6	124.7	26.2		56.2	7 (<10–34)	1.67 (<0.07–26.9)
DPTE	^1^ n.d.	^1^ n.d.	5.4	58.1	^1^ n.d.		31.7		
ATE	^1^ n.d.	^1^ n.d.	14.5	^1^ n.d.	^1^ n.d.		14.5		
HBB	^1^ n.d.	^1^ n.d.	28.3	24.2	138.2		63.6		0.36 (<0.003–2.11)
HCDBCO	32.0	^1^ n.d.	40.4	^1^ n.d.	^1^ n.d.	32.0	40.4		
PBBA	6.4	4.6	14.2	15.8	^1^ n.d.	5.5	15.0		
PBEB	<2.2	<2.2	^1^ n.d.	4.1	<2.2		4.1		0.06 (<0.003–0.25)
TBCO	^1^ n.d.	^1^ n.d.	<26.7	<26.7	40.7		40.7		
TBECH	41.8	33.7	38.3	41.5	228.7	37.7	102.8		
^2^ ΣnBFRs	301.4	566.2	239.9	33386.5	497.8	433.8	11,374.7		

Values between LOD and LOQ were considered positive and reported as <LOQ value. ^1^ n.d., not detected. ^2^ ΣnBFRs is the sum of nBFR concentrations.

**Table 5:** Parameters used for EDI_ingestion_ and EDI_dermal_ calculation.

Parameter		Reference
IR_dust_	60 mg/day	[46]
AF_gastro_ for BDEs and nBFRs	100%	[47,48]
AF_gastro_ for PCBs	85%	[49]
EF home	64%	[50]
EF workplace	22%	[50]
DAS	0.1 mg/cm^2^	[50]
BW	80 kg	[51]
ESA	4615 cm^2^	[52]

**Table 6:** List of AF_dermal_ factor used in this study.

Compound	Percent	Reference
BDE 28	27	[53]
BDE 47	33	[53]
BDE 49	33	[53]
BDE 66	33	[53]
BDE 85	34	[53]
BDE 99	34	[53]
BDE 100	34	[53]
BDE 153	37	[53]
BDE 154	37	[53]
BDE 183	37	[53]
BDE 197	8	[53]
BDE 209	8	[53]
TBBPA	40	[50]
TBPH	8	[53]
TBB	11	[53]
TBP	11	[53]
BTBPE	11	[54]
HBB	11	[45]
PBBA	34	
TBECH	27	[45]

**Table 7:** The sum ΣEDI_(ingestion+dermal)_ obtained for each different environment (D1–D5) is presented (Columns 2–6). The total contribution for each pollutant was calculated as ΣEDI PCBs, ΣEDI BDEs, and ΣEDI nBFRs. RfD is also reported. The values are expressed as ng/kg bw/day.

	D1	D2	D3	D4	D5	RfD [Ref]
	ΣEDI_(ingestion+dermal)_	ΣEDI_(ingestion+dermal)_	ΣEDI_(ingestion+dermal)_	ΣEDI_(ingestion+dermal)_	ΣEDI_(ingestion+dermal)_	
PCB 77 + 110			2.1		2.9	4.0 × 10^+1^ [55]
PCB 81	1.0	4.0 × 10^−1^		2.7 × 10^−1^		2.0 × 10^+1^ [55]
PCB 101			5.3 × 10^−1^			2.0 × 10^+1^ [55]
PCB 105					8.0 × 10^−1^	2.0 × 10^+1^ [55]
PCB 114	3.0 × 10^−1^	2.9 × 10^−1^				2.0 × 10^+1^ [55]
PCB 126					5.4 × 10^−2^	2.0 × 10^+1^ [55]
PCB 138		4.5 × 10^−1^	1.4 × 10^−1^	4.4 × 10^−1^	1.2	2.0 × 10^+1^ [55]
PCB 146				5.2 × 10^−2^	1.8 × 10^−1^	2.0 × 10^+1^ [55]
PCB 156		1.9 × 10^−1^		7.2 × 10^−2^	1.6 × 10^−1^	2.0 × 10^+1^ [55]
PCB 157		3.5 × 10^−1^			1.2 × 10^−1^	2.0 × 10^+1^ [55]
PCB 167		4.1 × 10^−1^			1.9 × 10^−1^	2.0 × 10^+1^ [55]
PCB 170 + 190	1.8 × 10^−1^	2.1 × 10^−1^		1.9 × 10^−1^	3.5 × 10^−1^	4.0 × 10^+1^ [55]
PCB 177		1.2 × 10^−1^		8.5 × 10^−2^	2.5 × 10^−1^	2.0 × 10^+1^ [55]
PCB 180	3.5 × 10^−1^	5.2 × 10^−1^	7.1 × 10^−2^	4.6 × 10^−1^	9.2 × 10^−1^	2.0 × 10^+1^ [55]
PCB 183	7.5 × 10^−1^	7.1 × 10^−1^		2.8 × 10^−1^	3.5 × 10^−1^	2.0 × 10^+1^ [55]
PCB 187				1.2 × 10^−1^	2.7 × 10^−1^	2.0 × 10^+1^ [55]
ΣEDI PCBs	2.6	3.6	2.9	2.0	7.7	
BDE 47	6.7 × 10^−1^	1.2	2.0 × 10^−1^	7.0 × 10^−1^	6.4 × 10^−1^	1.00 × 10^+2^ [56]
BDE 66			8.9 × 10^−1^			1.00 × 10^+2^ [56]
BDE 99	9.9 × 10^−1^	2.6	3.9 × 10^−1^	9.4 × 10^−1^	9.8 × 10^−1^	1.00 × 10^+2^ [56]
BDE 100	2.3	9.9 × 10^−1^	1.1 × 10^−1^		8.6 × 10^−1^	1.00 × 10^+2^ [48]
BDE 153			2.7 × 10^−1^			2.00 × 10^+2^ [56]
BDE 154		3.1 × 10^−1^	3.3 × 10^−2^		1.5 × 10^−1^	1.00 × 10^+1^ [48]
BDE 183		6.0 × 10^−1^	2.1 × 10^−1^	2.2	1.2	2.00 × 10^+2^ [56]
BDE 209					3.9 × 10^+1^	7.00 × 10^+3^ [56]
ΣEDI BDEs	4.0	5.7	2.1	3.9	4.3 × 10^+1^	
TBP	1.3	2.5	2.8 × 10^−1^	1.3 × 10^+1^	9.0 × 10^−1^	9.2 × 10^+4^ [55,57]
BTBPE			2.9 × 10^−1^	2.1	4.3 × 10^−1^	2.00 × 10^+4^ [58]
HBB			4.7 × 10^−1^	4.0 × 10^−1^	2.3	2.00 × 1^+3^ [33]
PBBA	3.1 × 10^−1^	2.2 × 10^−1^	2.4 × 10^−1^	2.6 × 10^−1^		
TBB	5.8 × 10^−1^	5.6 × 10^−1^		1.6 × 10^−1^	1.6 × 10^−1^	2.00 × 10^+4^ [58]
TBECH	2.0	1.6	6.4 × 10^−1^	6.9 × 10^−1^	3.8	6.8 × 10^+3^ [59]
TBPH	8.8	2.2 × 10^+1^	1.1			2.00 × 10^+4^ [60]
TBBPA				5.4 × 10^+2^		6.00 × 10^+5^ [61]
ΣEDI nBFRs	1.3 × 10^+1^	2.7 × 10^+1^	3.0	5.5 × 10^+2^	7.6	
